# Case Report: Potential Arsenic Toxicosis Secondary to Herbal Kelp Supplement

**DOI:** 10.1289/ehp.9495

**Published:** 2007-01-18

**Authors:** Eric Amster, Asheesh Tiwary, Marc B. Schenker

**Affiliations:** 1 School of Medicine; 2 School of Veterinary Medicine, University of California, Davis, California, USA; 3 California Animal Health & Food Safety Laboratory System, Davis, California, USA

**Keywords:** arsenic, DSHEA, heavy metal, herbal supplement, kelp, toxicity

## Abstract

**Context:**

Medicinal use of dietary herbal supplements can cause inadvertent arsenic toxicosis.

**Case Presentation:**

A 54-year-old woman was referred to the University of California, Davis, Occupational Medicine Clinic with a 2-year history of worsening alopecia and memory loss. She also reported having a rash, increasing fatigue, nausea, and vomiting, disabling her to the point where she could no longer work full-time. A thorough exposure history revealed that she took daily kelp supplements. A urine sample showed an arsenic level of 83.6 μg/g creatinine (normal < 50 μg/g creatinine). A sample from her kelp supplements contained 8.5 mg/kg (ppm) arsenic. Within weeks of discontinuing the supplements, her symptoms resolved and arsenic blood and urine levels were undetectable.

**Discussion:**

To evaluate the extent of arsenic contamination in commercially available kelp, we analyzed nine samples randomly obtained from local health food stores. Eight of the nine samples showed detectable levels of arsenic higher than the Food and Drug Administration tolerance level of 0.5 to 2 ppm for certain food products. None of the supplements contained information regarding the possibility of contamination with arsenic or other heavy metals. The 1994 Dietary Supplement Health and Education Act (DSHEA) has changed the way dietary herbal therapies are marketed and regulated in the United States. Less regulation of dietary herbal therapies will make inadvertent toxicities a more frequent occurrence.

**Relevance to Clinical Practice:**

Clinicians should be aware of the potential for heavy metal toxicity due to chronic use of dietary herbal supplements. Inquiring about use of dietary supplements is an important element of the medical history.

## Case Presentation

KB, a 54-year-old previously healthy woman, was referred to the University of California, Davis, Occupational and Environmental Medicine Clinic with a 2-year history of worsening alopecia and memory loss. Her symptoms began in October 2002 with minor memory loss and fatigue. Her primary care physician conducted a thorough physical exam, which showed no abnormality; all laboratory studies (complete blood count, electrolytes, thyroid-stimulating hormone) were also normal. KB’s physician believed her symptoms were related to menopause. With no specific diagnosis or treatment recommendations, our patient started taking a variety of herbal therapies including kelp supplement, fish oil, ginkgo biloba, and grape seed extract. The kelp supplement was the only herbal therapy taken regularly throughout the course of her illness. She initially took two tablets of Icelandic Kelp supplement (*Liminaria digitata*) per day (41 mg kelp, 66 mg calcium, 225 μg iodine). Over the next few months, she subjectively noticed new and progressively worsening symptoms. During her exam, it was noted that her short- and long-term memory had become impaired to the point where she could no longer remember her home address, a location where she had lived for the previous 5 years. She also began having difficulties functioning at work, where she did intake for a mental health facility.

KB’s gynecologist believed her symptoms were likely due to a relative estrogen deficiency and adjusted her estrogen dose. This resulted in no change in symptoms. Ancillary testing included a brain magnetic resonance image showing nonspecific changes. Further complete blood count, chemistry panel, and thyroid studies were also normal. Still lacking a clear diagnosis, KB decided to increase her daily kelp supplementation to at least four pills per day. At this point, she first noticed gastrointestinal (GI) symptoms including diarrhea, nausea, and vomiting without hematemesis. She developed daily pressure headaches as if “there was a band around her head.” Additionally, she became excessively weak and fatigued as the day progressed, requiring more sleep than previously. By the summer of 2003, she noticed a lacy, erythematous rash on her lower legs bilaterally. Other dermatologic findings included onchyolysis. Physical findings were confirmed on dermatologic assessment, which yielded a diagnosis of onchomycosis. An antifungal was prescribed but was of no benefit to her condition. By the summer of 2003, the debilitating nature of her symptoms forced her to leave her full-time job and take part-time work.

Her physician believed that her symptoms resembled those of a patient he had previously seen with arsenic toxicity. On 9 October 2003, a spot urine sample showed an arsenic concentration of 83.6 μg/g creatinine (normal < 50 μg/g creatinine). No lead or mercury was detected.

A detailed exposure history focusing on potential sources of arsenic was conducted. KB’s home water is from a deep well that was found to contain arsenic below detectable levels. Her diet consisted of less than one serving of seafood/fish per week. A sample of the kelp supplement was analyzed by Columbia Analytic Services (Kelso, WA) and showed an arsenic concentration of 8.5 mg/kg. No arsenic was detected in the fish oil she had been taking. The ginkgo biloba and grape seed extract were not analyzed.

Given the arsenic contamination in the kelp supplement, her physician recommended that she stop taking the supplement. She noted a dramatic improvement in her neurologic, GI, and dermatologic symptoms. Three weeks after stopping supplement use, KB returned to full-time work on 1 November 2003, with nearly complete resolution of symptoms. Repeat spot urine arsenic on 10 December 2003, was 25 μg/L (normal 0–50 μg/L), down over one-third from 2 months before. In a urine sample analyzed on 26 February 2004, arsenic was undetectable. Blood arsenic on 26 February 2004, was 8 μg/L (normal for reporting laboratory, 2–23).

## Discussion

### Clinical manifestations of chronic arsenic exposure

Chronic arsenic toxicity may cause peripheral neuropathies, parasthesia, ataxia, cognitive deficits, fatigue, and muscular weakness. GI complaints include anorexia, hepatomegaly, jaundice, nausea, and vomiting. Skin afflictions may include erythema, eczema, pigmentation (arsenic melanosis), diffuse alopecia, keratosis (especially of palms and soles), scaling and desquamation, brittle nails, white lines or bands in the nails (Mees lines), and localized subcutaneous edema. White striae in the fingernails are consistent with a diagnosis of arsenical polyneuritis, even though urine and hair arsenic concentrations may be within normal limits (Heyman et al. 1956).

Manifestations of chronic arsenic ingestion depend on both the intensity and duration of exposure. Our case had a more severe presentation than would be expected with an elevated urinary arsenic concentration of 83.6 μg/g creatinine. The intensity of her symptoms may have been the result of her lengthy duration of exposure or perhaps of an undiagnosed underlying condition. Also, that a single spot urine may not be as accurate as a 24-hr urine sample, despite adjustment for creatinine concentration.

In most cases the toxic moiety is presumably trivalent arsenic in the form of inorganic arsenious acid (arsenite), or an organic arsenoxide, rather than the element itself. Pentavalent arsenicals may be reduced, to a small extent *in vivo*, to the active trivalent form. This *in vivo* conversion may explain why all chemical forms of arsenic eventually produce the same toxic syndrome.

### Dietary sources of arsenic

Elemental arsenic is found naturally in the earth’s crust at concentrations of 2–5 ppm (Tamaki and Frankenberger 1992). Arsenic is released into the environment through both natural sources (i.e., soil erosion, volcanoes) as well as anthropogenic sources (e.g., release from metal mining and smelting, pesticide application, coal combustion, waste incineration). Most arsenic release into the environment is inorganic and accumulates by binding to organic soil matter (Smedley and Kinniburgh 2005).

Soils with high arsenic concentrations can yield foods with exceedingly elevated arsenic levels. Diet is the largest source of exposure for nonoccupationally exposed individuals, with an average total (inorganic and methylated) arsenic intake of 40 μg/day. U.S. dietary intake of inorganic arsenic has been estimated to range from 1 to 20 μg/day (Schoof et al. 1999). Because of high arsenic concentration in algae and marine micro-organisms, seafood is the highest dietary source of arsenic (Tao and Bolger 1999). Arsenic concentrations for fish and seafood average 4–5 ppm (Bennett 1981), significantly higher than concentrations found in grains and cereals, with an average of 0.02 ppm (Gartrell et al. 1986). Although chronic low-level exposure to arsenic does occur from dietary sources, it is usually significantly below toxic levels. The tragedy of acute and chronic arsenic poisoning from contamination of water in Bangladesh, West Bengal, and elsewhere in the world has recently been described (Mead 2005).

### Homeopathic medications and arsenic toxicity

A number of published studies have highlighted cases in which homeopathic remedies cause clinical arsenic toxicity. One such study describes 74 patients in Singapore who were victims of chronic arsenic poisoning caused by local antiasthmatic herbal preparations (Tay and Seah 1975). Systemic involvement mainly affected the patients’ skin (hyperpigmentation, hyperkeratosis), nervous system (polyneuropathy, tremors, headache), and GI system (gastroenteritis, toxic hepatitis). Of the 74 patients studied, 10 presented with malignancies. Of the 29 herbal preparations analyzed in this study, 16 contained inorganic arsenic in concentrations of 25–1,000 ppm, and 10 contained 1,001–50,000 ppm.

Mitchell-Heggs et al. (1990) reported on a 33-year-old Korean woman who presented with malaise, difficulty walking, arthralgia, and diarrhea. Her elevated urine and blood arsenic levels were linked to an herbal treatment for hemorrhoids, which contained 10,000 ppm arsenic. The patient recovered completely after ceasing her usual dosage of 90 pills/day (50 mg/day).

Espinoza et al. (1995) analyzed traditional Chinese herbal balls, which are taken for a variety of conditions, including rheumatism and cataracts. The herbal balls were found to contain up to 36.6 mg arsenic per ball. The authors concluded that with a “recommended dose” of two herbal balls daily, the preparation “poses a potentially serious health risk to consumers.” They advised that “health professionals should be aware that patients who consume traditional Chinese remedies may be exposed to potentially toxic substances” (Espinoza et al. 1995).

To our knowledge, only one case study has previously documented arsenic toxicity related to consumption of herbal kelp supplements. Walkiw and Douglas (1975) reported on two patients admitted for neurologic investigation with elevated urinary arsenic excretion (138 and 293 μg/24 hr). Through detailed history and laboratory sampling, both cases were linked to ingestion of kelp supplement. Urinary arsenic concentrations declined to normal range (< 10 μg/24 hr) within 3 months of discontinuing kelp supplements. The initial presenting signs of foot-drop also resolved.

### Laboratory data

To assess the concentration of arsenic present in commercially available kelp supplements, we purchased nine over-the-counter kelp samples from local health food outlets. Included were samples from three different batches of the product that was consumed by the patient. We determined total arsenic in the herbal samples by inductively coupled argon plasma (ICP) using the identical hydride vapor generation method, as described by Tracy et al. (1991). The ICP used was an Applied Research Laboratories–model Accuris, and fitted with a Noordermeer V-groove nebulizer (both from Fisons Instruments, Valencia, CA). All samples were analyzed randomly in a blindfolded manner.

Laboratory analysis by the California Animal Health & Food Safety Laboratory found detectable levels of arsenic in eight of the nine kelp herbal supplements, ranging from 1.59 ppm to 65.5 ppm by dry weight (1.59, 2.28, 9.55, 9.97, 10.5, 24.1, 34.8, and 65.5 ppm); the median value was 10.23 ppm. One of the nine samples was below the method detection limit of 0.010 ppm. The three samples of the brand of kelp supplement consumed by our patient throughout the duration of her symptoms showed arsenic concentrations of 34.8, 2.28, and 1.59 ppm.

In a recent analysis of ayurvedic herbal medicine products, Saper et al. (2004) found that 20% of products tested contained heavy metals. Detectable arsenic levels in 6 of 70 samples ranged in concentrations from 37 ppm to 8,130 ppm. Although the kelp samples we analyzed were consistently elevated, the concentration of arsenic in our samples was considerably lower than previously documented concentrations in herbal remedies (Mitchell-Heggs et al. 1990; Saper et al. 2004; Tay and Seah 1975). This raises the concern that chronic exposure to contaminated supplements, even with moderately elevated arsenic concentrations, could still be toxic. None of the supplements contained labeling information regarding the possibility of contamination with arsenic or other heavy metals.

The Food and Drug Administration (FDA) has set tolerance levels for arsenic in certain food products. These permissible levels range from 0.5 ppm in eggs and uncooked edible tissues of chickens and turkeys to 2 ppm in certain uncooked edible by-products of swine. The concentration of arsenic found in seven of the nine supplements exceeded the FDA tolerance level of 2 ppm (Agency for Toxic Substances and Disease Registry 2006).

### Regulation and standards of homeopathic remedies

In 1998, the California Department of Health reported that 32% of traditional Asian medicines sold in the state contained heavy metals (including lead, mercury, and arsenic) or undeclared pharmaceuticals (Ko 1998). Although this case report focuses on the potential for arsenic contamination in herbal supplements, lead exposure from supplements is also of increasing concern (Mattos 2006). Labeling information provides little warning; two-thirds of homeopathic medicines sampled contained arsenic levels higher than indicated on the label (Kerr and Saryan 1986).

The popularity of herbal treatments and dietary supplements has increased at an astonishing rate. In 2001, $178 billion was spent in the United States on dietary supplements (Anonymous 2002). Of the many reasons for the increasing popularity of supplements, the most important has been attributed to the enactment of the Dietary Supplement and Health Education Act (DSHEA 1994; Marcus and Grollman 2002). DSHEA amends the Food and Drug Act (1938) to define the way dietary supplements are regulated and labeled. Under DSHEA (1994), herbal, vitamin, and mineral treatments are considered dietary supplements, and the act limits the FDA’s control over substances classified as dietary supplements.

Commenting on DSHEA, former FDA commissioner David A. Kessler stated that

[T]he Act does not require that dietary supplements be shown to be safe or effective before they are marketed. The FDA does not scrutinize a dietary supplement before it enters the marketplace. (Kessler 2000)

Instead, Kessler noted, the FDA must rely on adverse event reports to determine product safety and efficaciousness. As a result, dietary supplements such as herbal therapies are now subject to lower safety standards than food additives. In a review of regulations for botanical medicines, Mitchell-Heggs et al. (1990) warned that

Consumers are provided with more information about the composition and nutritional value of a loaf of bread than about the ingredients and potential hazards of botanical medicines.

## Conclusion

Heavy metal intoxication should be suspected in a patient presenting with idiopathic neuropathy. When neuropathy is accompanied by an elevated urinary arsenic level, it is crucial that the physician determine the source of the arsenic (Walkiw and Douglas 1975). Our patient, KB, had complete resolution of her symptoms and was able to return to work within weeks of discontinuing her kelp supplements. Her urine arsenic levels returned to normal within 2 months of the initial diagnosis. Given the nature of her symptoms and the temporal association of her kelp ingestion with the occurrence and resolution of the symptoms (an association supported by urinary arsenic levels, which were elevated as her kelp ingestion increased), we believe that there was a causal association between her ingestion and symptoms. There was also clear biological plausibility in this case; arsenic is known to cause the symptoms observed in our patient, and previous studies have demonstrated toxicity from intake of dietary herbal supplements. We were unable to identify any other probable dietary or environmental exposure. We recognize, however, that the estimated dose of her exposure in this case was based on limited analyses, and was therefore approximate.

Our finding that eight of the nine commercially available kelp supplements contained detectable levels of arsenic is a cause for concern. It appears from our results that there is little consistency in the arsenic content of the kelp supplements from batch to batch. Three samples, all the same brand, showed variability in contamination ranging from 1.59 ppm to 34.8 ppm arsenic. It is unlikely that people are aware of the potential exposures they receive from herbal supplements. Not one of these products had labels indicating the possibility of arsenic or other heavy metals in the kelp. It is unfortunate that a therapy which is advertised (on the label) as contributing to “vital living and well-being” would have potentially unsafe levels of arsenic.

Given the numerous studies demonstrating unsafe levels of heavy metals in dietary herbal preparations, the growing number of case reports connecting heavy metal toxicities to ingestion of herbal dietary supplements, and the growing popularity of herbal remedies for self-medication in the general public, it is prudent that companies demonstrate safety and efficacy before their products are placed on the market. Concentrations of materials contained in the preparations, as well as expected benefits and potential side-effects, should be studied, standardized, monitored, and accurately labeled.

## Correction

In their discussion of the variation of arsenic content of kelp supplements from batch to batch in the conclusion of the original manuscript published online, the authors stated that three samples they tested were all from the same bottle. However, the samples were all the same brand but not from the same bottle. The error has been corrected here.
